# Neutrophil Extracellular Traps (NETs) and Atherosclerosis: Does Hypolipidemic Treatment Have an Effect?

**DOI:** 10.3390/jcdd11030072

**Published:** 2024-02-21

**Authors:** Petros Spyridonas Adamidis, Despoina Pantazi, Iraklis C. Moschonas, Evangelos Liberopoulos, Alexandros D. Tselepis

**Affiliations:** 1 Department of Internal Medicine, Faculty of Medicine, School of Health Sciences, University of Ioannina, 45110 Ioannina, Greece; padam7@yahoo.gr; 2 Atherothrombosis Research Centre, Laboratory of Biochemistry, Department of Chemistry, University of Ioannina, 45110 Ioannina, Greece; dpantazi@uoi.gr (D.P.); iraklismoschonas@hotmail.com (I.C.M.); 3 1st Propaedeutic Department of Medicine, School of Medicine, National and Kapodistrian University of Athens, Laiko General Hospital, 11527 Athens, Greece; vaglimp@yahoo.com

**Keywords:** atherosclerosis, dyslipidemia, lipid-lowering therapies, neutrophils, neutrophil extracellular traps

## Abstract

Neutrophil extracellular traps (NETs) have attracted much attention recently, beyond elemental host immunity, due to their fundamental implication in a variety of pathologic conditions and widespread impactful diseases. Atherosclerotic cardiovascular disease (ASCVD) is one of them, and a major cause of mortality and disability worldwide. Consequently, years of basic and clinical research were dedicated to shedding light on every possible pathophysiologic mechanism that could be used as an effective prevention and treatment tool to ameliorate its burden. This led to the development of complex and prevention protocols and regimens that are now widely used, with lipid-lowering treatment being the current cornerstone; however, this is not adequate to alleviate the residual cardiovascular risk, which remains prominent. Despite the demonstrated pathogenic role of NETs in the progression and complications of ASCVD, little is known about their potential as a therapeutic target and the effects hypolipidemics exert on them.

## 1. Introduction

Extracellular traps (ETs) are large, web-like structures composed of decondensed DNA, and the process of ET formation is known as ETosis [1]. While neutrophil ETtosis (NETosis) was initially used to describe a new form of neutrophil death, different from apoptosis, necrosis, and necroptosis [1], evidence of ETs has also been reported for macrophages, eosinophils, basophils, mast cells, and dendritic cells [2]. Although neutrophil extracellular traps (NETs) are thought to be an evolutionary, conserved defensive element due to their protective abilities like trapping, killing, and restraining microorganisms, it is their vast pathogenic potential that has recently emerged as a research topic of great interest [3,4]. Netosis dysregulation is well documented as being implicated in the pathogenesis and negative course of a number of diseases falling into the cardiovascular, autoimmune, autoinflammatory, metabolic, infectious, and neoplastic spectra [3,4]. Some microbes can evade NETs’ host-protective barrier, while others have the ability to utilize the barrier for their own host-damaging purposes [3,4,5,6,7,8,9]. Taking into account the dysregulated overproduction of NETs, they may become the source of severe tissue damage in conditions like sepsis and COVID-19 [3,4,5,6,7,8,9]. Providing a mechanical scaffold and interfering with the intrinsic and extrinsic coagulation cascade, as well as platelets, NETs formed in the circulation promote arterial and venous thrombosis [4,10,11,12]. Moreover, footprints of NET formation, like citrullinated histones, cell-free DNA, and MPO–DNA complexes, are found to be elevated in the plasma and tissue of patients with abdominal aortic aneurysms [3,13,14,15]. Through various and complex mechanisms and disease-specific triggers, neutrophils and their NETs are fundamentally implicated in the pathophysiology of atherosclerosis, diabetes, vasculitis, inflammatory bowel disease, cancer metastases, Alzheimer’s disease, different types of arthritis, systematic lupus erythematous, and obesity [3,4,16].

Diving deeper into the study of atherosclerosis, it is worth mentioning that atherosclerotic cardiovascular disease (ASCVD) remains a major worldwide cause of mortality [17]. The prolongation of life expectancy, in concurrence with advancements in modern medicine, has allowed many people to experience the negative outcomes of an unhealthy habitual lifestyle, which are expressed via chronic conditions that take time to evolve and, if not abruptly terminate, certainly diminish patients’ quality of life [18]. Atherosclerosis, one of these conditions, evolves insidiously, beginning from early childhood [19], and leads to most cardiovascular diseases. The two fundamental aspects of the initiation and progression of atherosclerosis are lipoproteins and inflammation [20]. However, in order to harm the vessel, these factors need the lucrative substrate of endothelial dysfunction, consequently leading to an increase in the permeability and expression of cytokines [21]. Hypercholesterolemia, hypertension, insulin resistance, elevated lipoprotein(a) [Lp(a)], the use of tobacco, the accumulation of visceral adipose tissue, and diabetes are major proatherogenic risk factors that have been identified and well-studied due to their causal role in atherogenesis, provoking endothelial dysfunction possibly via increased oxidative stress [21]. Nonetheless, the complex underlying molecular pathways are still not fully elucidated [20,21]. Prolonged exposure to increased LDL-C particles is undoubtedly associated with the initiation of atherosclerosis [18]. In addition, increased triglyceride-rich lipoproteins (TGRLs), in conjunction with low high-density lipoprotein (HDL), play an important role in atherogenesis [22].

The introduction of statins has revolutionized the prevention and treatment of atherosclerosis [18]. Statins have a 30–50% LDL-C reduction efficacy. This is increased when statins are combined with ezetimibe (15–20%) and the inhibitors of PCSK9 (PCSK9i) (50–60%) [23]. Beyond LDL-C, new treatments targeting triglyceride-rich lipoproteins and Lp(a) have become available or entered clinical development. The potential mechanisms of action of current and emerging Lp(a)-lowering therapies have recently been reviewed [24]. Biological and RNA-directed agents have joined traditional small-molecule approaches while gene-editing approaches have appeared on the horizon of lipid management [23].

Despite the optimal management of traditional risk factors according to current treatment guidelines, a significant residual cardiovascular risk still exists [25]. Therefore, it is important to explore every aspect of atherosclerosis as a potential therapeutic target and meticulously study every relevant treatment effect. Inflammation, being a cornerstone of atherosclerotic procedure [26], remains undoubtedly an attractive field. In this context, we researched the potential effects of lipid-lowering medications on NETs and NETosis. Already published review articles by Soehnlein et al. [26], Libby et al. [18,20], and Weber et al. [27] have been enlightening and inspiring for more research on the topics of atherosclerosis, immunity, and inflammation. Our review aims to provide a brief and concise summary of their highlights, among others, and also a summary of the current literature on the specific effects of widely-used hypolipidemic treatment on NETs and NETosis to aid the investigation of their pleotropic actions and motivate more research on the topic.

## 2. Neutrophil Extracellular Traps (NETs)

### 2.1. Definition and Formation

NETs, as their acronym suggests, are web-like structures excreted by neutrophils upon activation by various stimuli. The process of their formation and release is called NETosis [28]. They consist of nuclear and occasionally mitochondrial chromatin, which is decondensed by cytosolic enzymes to form the NET’s fibers. These long DNA fibers were revealed by high-resolution scanning electron microscopy to have diameters of 15–17 nm and globular domains of 25–50 nm aggregates. They contain proteins found in neutrophil granules, like myeloperoxidase (MPO), neutrophil elastase (NE), cathepsin G, and gelatinase. These, along with chromatin histone cytotoxic effect, grant NETs a number of toxic and dissemination-hindering properties against various pathogens [3,4,29]. NETs also gather proteins from their surroundings, with tissue factor being one of them [10]. Concerning protein content, NETs are not homogenous, implying the varying origin from different neutrophil subpopulations [30,31]. The resulting web-like structure has a volume of 10–15 times larger than that of the originating cell [3,32].

As mentioned, NETs are created and released via a process of cell death distinct from apoptosis, necrosis, and necroptosis, generally referred to as NETosis [1,30,33]. There are two types of NETosis. The first, named lytic NETosis, includes delobulation of the nucleus, disassembly of the nuclear envelope, decondensation of chromatin, mixing with cytosolic and granule proteins, loss of cellular membrane polarization, rupture of cellular membrane, and, finally, release of NETs. This takes place 3–8 h upon neutrophil activation. The claim that NETosis is a type of programmed cell death is supported by the fact that membrane permeabilization happens in a programmed manner and not as a physical consequence of chromatin expansion [4,34,35]. The second type of NETosis is a more rapid one observed within minutes of exposure to Staphylococcus aureus and does not involve cell destruction. It seems that nuclear chromatin secretion occurs in parallel with degranulation of granule proteins and extracellular assembly of the previously described final net structure. This process leaves behind anucleated cytoplasts that maintain the ability to phagocytose bacteria and is observed in the first neutrophils to arrive at sites of infection [4,34,35].

### 2.2. Triggers

Several stimuli have been reported to trigger NETosis and modulate its components. Microorganisms induce NETosis depending on their size, virulence factors, and released inflammatory molecules [3]. NETs have been reported to effectively combat bacteria [4,28], viruses [4,36], parasites [4,37], and fungi [4,38]. Smaller microbes are usually phagocytosed by neutrophils, while larger ones or those that form aggregates block this mechanism and are, thus, subjected to NETosis [3,4]. Other endogenous and exogenous stimuli that trigger NETosis are damage-associated molecular patterns (DAMPs), immune complexes, nitric oxide, urate and cholesterol crystals, autoantibodies, proinflammatory cytokines, and interactions between neutrophils and platelets or neutrophils and endothelial cells [3,4,39,40]. The size of the non-septic stimuli also contributes to the formation of NETs, with larger urate crystals, for example, being a more drastic trigger than smaller ones [3,4,39,40]. Each of the various stimuli initiates the process of NETosis via different plasma membrane receptors and downstream molecular pathways, implicating nicotinamide adenine dinucleotide phosphate (NADPH), reactive oxygen species (ROS), myeloperoxidase (MPO), neutrophil elastase (NE), and protein arginine deiminase (PAD) enzymes [4]. However, in vivo, the exact participation of each pathway remains obscure. The cooperative and parallel activation of more than one NETosis-conducting mechanism is the most probable scenario [3,14,41,42,43,44]. Interestingly, the differences in NETs structural characteristics, such as variable histone citrullination by different stimuli, could help identify the involved immunopathogenic mechanism in vivo [4]. Moreover, recent research suggests that neutrophil circadian rhythmicity applies to NETs formation [10,45].

### 2.3. NETs Content and Quantification

NETs contain proteins found in neutrophil granules, like MPO, NE, cathepsin G, and gelatinase. MPO has the potential to modulate the oxidation of LDL [46]. The MPO–DNA complex is considered the most specific, objective, and quantitative plasma marker for NETs formation [47]. Other forms of NET remnants are complexes of DNA and NE (NE–DNA) [47]. Citrullinated histone 3 (CitH3) is another widely used marker for NETs formation [47]. MPO–DNA and NE–DNA complexes in fluid samples can be determined by enzyme-linked immunosorbent assay (ELISA) [47]. Although problems of standardization exist, this methodology remains the most used for monitoring NETosis [47]. As mentioned, NETs can gather proteins from their surroundings, with tissue factor (TF) being one of them [10].

## 3. Correlation of NETs to Atherosclerosis and Implication in Its Pathogenesis

### 3.1. Atherosclerosis, Immunity and Inflammation

The recent advancements in understanding molecular and cellular mechanisms in atherosclerosis, as well as future perspectives, have been described [2]. Accumulating evidence suggests that inflammation is the key component linking risk factors with atherosclerosis [18]. While oxidized LDL particles are well-studied drivers of atherogenesis [18,48], TGRLs are correlated to inflammatory status more effectively than LDL particles [49,50], as reflected by levels of high-sensitivity C-reactive protein (hsCRP) [51]. Consistent links are also documented with hypertension [52], obesity [53], and diabetes [54]. Importantly, many studies document the participation of innate and adaptive immunity to atherosclerosis pathophysiologically [55] and as a promising therapeutic target [18].

Under physiological conditions, macrophages reside in the vascular cell wall, specifically in the adventitia or under the endothelium, where they contribute to the maintenance of vascular homeostasis by interacting with SMCs and endothelium [26,56,57]. Endothelial cells damaged by well-studied stimuli like hypercholesterolemia, hypertension, diabetes, and oxidative stress attract monocytes with the contribution of activated platelets, by expressing leukocyte adhesion molecules like VCAM-1 [18,27] and excreting chemokines and guide them in the vascular wall intima via expression of leukocyte adhesion molecules, mostly integrins, on their surface. This marks the beginning of atheroma formation. Monocyte/macrophage recruitment and local proliferation make them the cornerstone of the atherosclerotic process [26]. Neutrophils and activated SMCs aid the monocyte infiltration by excreting chemokines like cathelicidin, cathepsin G, CCL2, and CCL5 [26]. SMCs also migrate to the developing fibrous cap and undergo apoptosis there after their metaplasia to SMC foam cells induced by lipid uptake. Macrophages also uptake lipids and transform into foam cells that comprise the lipid core of atherosclerotic plaque [18,58]. This uptake, particularly of oxidized LDL [59], along with reduced cholesterol efflux [60], triggers the activation of inflammasome NLRP3, which, in turn, leads to maturation of IL-1β and IL-18 [26,59]. Neutrophils also excrete their NETs, which enhance inflammasome priming and exert cytotoxic effects on SMCs via histone H4 [26]. Conversely, inflammasome activation also causes the production of NETs via IL-18, as documented here [61]. The fundamental role of inflammasome in the atherosclerotic process is underlined by a study that documented improvement in plaque stability after either genetic or pharmacologic inhibition of absent in melanoma 2 (AIM2), a DNA-sensing cytosolic part of the inflammasome [62]. Activated T-helper1 lymphocytes also co-orchestrate and propagate the fluctuating imbalance of proinflammatory and anti-inflammatory molecules that eventually, through years of process, lead to atherosclerosis [18]. Ultimately, atherosclerosis appears to be the result of failure to counteract the aforementioned inflammatory mediators by their counterpart anti-inflammatory molecules excreted by B1, T-helper 2, and regulatory T lymphocytes like IL-10 and TGFβ [18]. Notably, inadequate clearance of cellular debris and dying cells by mononuclear phagocytes, a process called efferocytosis, leads to their accumulation and the formation of the lipid core of the atherosclerotic plaque [18].

### 3.2. Complications Leading to ASCVD Events

Rupture, superficial erosion, and increase in size of the atherosclerotic plaque become clinically apparent as cardiovascular disease (CVD) events [18]. In the past, the major mechanism for plaque disruption was thought to be the thinning and rupturing of the fibrous cap of the plaque due to collagen degradation by ongoing inflammation [63]. This results in exposing thrombogenic components to the circulation, priming the coagulation cascade, and leading to concurrent thrombotic events [64]. However, imaging studies have demonstrated that the most vulnerable, thin-capped plaques are the least clinically overt [18,65,66,67]. The most likely mechanism causing plaque vulnerability and concurrent CVD events appears to be the superficial erosion of the plaque, with neutrophils and their extracellular traps being the major components initiating and propagating this process [68,69,70].

### 3.3. The Role of NETs in Atherosclerosis

As previously indicated, the atherosclerotic process is grounded on an intricate interplay between vascular homeostasis and the immune system. Research underscores the pivotal role of neutrophils and NETs throughout every phase of the atherosclerosis timeline, from initiation to the clinically evident thrombotic complications [10]. Initial indications of NETs’ involvement in atherosclerosis surfaced through experimental investigations on plaques derived from mice and humans [71]. Apolipoprotein E deficient mice were subjected to either a high-fat or a high-fiber diet for four weeks and atherosclerotic plaques derived from both groups were analyzed afterwards. The presence of luminally adhered neutrophils excreting NETs was determined in 57% of the first group’s atherosclerotic lesions while no neutrophils were observed in the second group specimens [71]. Similar findings were replicated in human atherosclerotic plaques post-carotid endarterectomy within the same study [71]. Subsequent research documented neutrophil and NETs abundance mostly in complicated plaques either with thrombosis or rupture [72]. Among 64 autopsy-derived specimens from post-myocardial infarction patients, 44 contained complicated and 20 intact atherosclerotic plaques. Neutrophils and NETs were predominantly observed in complicated plaques with ruptures, erosions, and intraplaque hemorrhage (*p* < 0.05) in similar amounts at each complication type and mostly in early stages of thrombus formation and plaque hemorrhage [72]. This observation was corroborated by the finding that NETs’ histone H4 favors inflammatory process and plaque destabilization by affecting smooth muscle cells. In this study, both atherosclerotic mouse models, as well as human endarterectomy samples, were employed to investigate the connection and participation of neutrophils and NETs in this context. Overall plaque vulnerability was significant in neutrophilic but not neutropenic mice [73]. Moreover, observations of the exact location where neutrophils are concentrated and of their interactions with smooth-muscle cells were documented. In summary, the well-designed experiments demonstrated that activated smooth-muscle cells attracted neutrophils on-site through excretion of chemokines, particularly CCL7, as well as ROS production by SMCs, triggering significant NETs release. CCL7 blockade resulted in reduced NETosis [73]. On the other hand, NETs exhibited strong cytotoxic effect on SMCs, mostly via H4 histone, resulting in reduced amounts of the latter in atherosclerotic lesions and increased plaque vulnerability due to thinning of plaque’s fibrous cap [26,73]. Notably, extranuclear histone H4 showed a significant positive correlation with intimal neutrophil counts. When the researchers neutralized H4 histone using specific antibodies, SMCs numbers and plaque stability remained intact [73]. Similarly, when they blocked NETosis using knockout mice for the PAD4 enzyme or through pharmacologic inhibition using chloramidine, plaque stability was preserved [73]. In another elegantly designed series of experiments, the induction of ROS-dependent NETosis by cholesterol crystals was demonstrated [74]. Nicotinamide adenine dinucleotide phosphate (NADPH) oxidase and NE, fundamental in ROS-dependent NETosis, inhibition efficiently blocked cholesterol-induced NETosis, specifically while cloramidine (a PAD4 inhibitor) failed, obviously due to different NETosis molecular pathways [74]. Consequently, researchers employed mouse models of atherosclerosis to elaborate on the roles of neutrophils and NETs in the process. They noted that ApoE-deficient mice after DNase injection and ApoE/PR3/NE-deficient mice, namely incapable of producing or using NETs, experienced a significant reduction in atherosclerotic lesion size after eight weeks of a high-fat diet, compared to control ApoE-deficient counterparts without any treatment [74]. Moreover, NETosis-inactivated models demonstrated lower levels of circulating cytokines in the same time period. Notably, DNase administration caused a reduction in plasma cytokines in ApoE-deficient but not in ApoE/PR3/NE-deficient mice where interleukines (IL) IL-1α,-1β,-6 were already absent [74]. In particular IL-1β, a fundamental cytokine triggered by activated macrophages to recruit neutrophils, was significantly reduced to being absent in the latter model lesions. Naturally, cytokine regulation by NETs affects all immune cell communication, and that is also documented hitherto [74]. Monocytes exposed to supernatants containing NETs were more sensitive to cholesterol stimulation and produced larger amounts of cytokines [74]. Finally, IL-1β-regulated T-cells, which also promote neutrophil recruitment, and total immune cell counts in atherosclerotic lesions were also significantly less in ApoE/PR3/NE-deficient mice than ApoE-deficient controls. Thus, the aforementioned study [74] proved the NETs priming and amplifying effect in the complex cellular interplay between macrophages, neutrophils, and T-cells in the setting of atherosclerosis. PAD4, a fundamental enzyme for histone citrullination and chromatin decondensation [75], has successfully been targeted by chloramidine, thus inhibiting NETs release in atherosclerotic murine models and alleviating atherosclerosis by decreasing lesion size [26,76]. However, as clearly stated here [26], chloramidine’s incapability of targeted PAD4 isoform inhibition renders it unsuitable for clinical use. In a model of PAD4 and ApoE-deficient mice atherosclerosis burden was diminished in accordance with reduced inflammatory status and NETs formation [77]. Interestingly, PAD4 deletion in a murine model of LDLR-deficient animals failed to improve plaque size or composition after ten weeks of a high-fat diet, despite documented limited NETosis on-site. However, it benefited plaque stability by reducing intimal injury and thrombus formation [75]. Furthermore, in the same study, NET components like NE and citH4 were localized vastly in superficial erosion plaques compared to rupture-prone ones in human samples derived from endarterectomy procedures, implying NETs involvement in the specific type of plaque complication [75]. Importantly, NETosis has been triggered also by stimuli and pathways that do not implicate PAD4 in the process [78], meaning that PAD4 inhibition alone might not cause sufficient NETosis suppression in clinical practice.

There is also a considerable amount of clinical evidence on NETosis engagement in atherosclerosis, which has been concisely summarized recently by Doring et al. [10]. For instance, in a prospective, observational, cross-sectional cohort study of 282 patients with possible coronary artery disease (CAD), increased NETosis biomarkers-dsDNA, nucleosomes, and MPO–DNA complexes were identified in the plasma of individuals with severe coronary atherosclerosis compared to those without significant coronary disease [43]. Nucleosomes emerged as an independent marker of severe coronary stenosis (OR = 2.14, 95% CI 1.26–3.63; *p* = 0.005), while MPO–DNA complexes predicted major CVD events during the study [43]. Moreover, dsDNA levels were higher in those with severe (CAD) (*p* = 0.003) or increased coronary artery calcification (*p* < 0.001) compared with patients without CAD [43]. Luminal stenosis was also positively associated with circulating dsDNA (Spearman’s ρ = 0.271; *p* < 0.001) and the number of pathological coronary artery segments with plasma dsDNA (Spearman’s ρ = 0.242; *p* < 0.001), nucleosomes (Spearman’s ρ = 0.219; *p* = 0.001), and MPO–DNA complexes (Spearman’s ρ = 0.337; *p* < 0.001) [43]. Importantly, baseline levels of the aforementioned circulating NETosis biomarkers emerged as sufficient predictive tools for the occurrence of major adverse cardiovascular events (MACE) during a median follow-up period of 545 days [43]. Finally, NETs are believed to be a new source of TF in atherothrombosis [79]. A plethora of evidence implicating NETs in thrombotic complications following atherosclerosis has been analyzed here [30].

## 4. Lipid-Lowering Treatment and NETs

### 4.1. Current Knowledge on Lipid-Lowering Treatment

Statins and their combination with ezetimibe stand as the most frequently prescribed drugs for lowering LDL-C. Additionally, proprotein convertase subtilisin/kexin type 9 (PCSK9) inhibitors find use in the management of hypercholesterolemia. Bempedoic acid, an ATP citrate lyase (ACLY enzyme) inhibitor, effectively reduces LDL-C (by 17–28%), presenting a viable alternative without the heightened risk of the musculoskeletal adverse effects associated with statins. Hypertriglyceridemia is currently treated by icosapent ethyl (IPE), a highly purified formulation of eicosapentaenoic acid (EPA), in high-risk patients. Furthermore, elevated TG can also be mitigated by fibrates particularly for pancreatitis prevention.

### 4.2. Documented Effects of Lipid-Lowering Treatment on NETs and NETosis

Since the discovery of the implication of NETs in numerous pathologies, many of their components, and their structure as well, have emerged as potential therapeutic targets. As previously mentioned, PAD4 has been an appealing target, yet, so far, the inhibitory methods lack specificity, which is necessary for clinical use. On the contrary, the first antibody engineered to possess NET-inhibiting properties is a therapeutic anti-citrullinated protein antibody (tACPA) and has shown promising results in preclinical models of murine and human NETs [80]. Moreover, a DNA-dissolving agent, namely DNase, has undergone testing in murine atherosclerotic lesions, where it appeared to have stabilizing effects [73]. Likewise, DNase administration within 6 hours after myocardial infarction in mice resulted in favorable outcomes regarding cardiomyocyte survival and ventricular remodeling [81]. Furthermore, co-administration of DNase with recombinant tissue-type plasminogen activator (rt-PA) reduced NET density and significantly alleviated the ischemic aftermath, namely ischemic area dimensions, left ventricular remodeling, and infarct size in another murine model [82].

The impact of lipid-lowering agents on NETs has not been adequately addressed. Few studies evaluated the effects of statin treatment on NETs and NETosis in vivo mostly in murine models, yielding contradictory results.

Atorvastatin, in comparison to PBS, induced significant reduction (*p* < 0.05) in neutrophil and citrullinated histone H3 (CitH3) levels present in murine venous thrombi on day 4 of treatment [83]. Moreover, in another murine model of thermal injury, simvastatin exerted a significant protective effect against post-injury inflammation and systemic NETosis [84]. Conversely, a study has reported in-vitro-enhanced NETs production in human neutrophils treated with mevastatin, lovastatin, fluvastatin, and simvastatin compared with control [85]. The authors reasonably deduced through their analysis that this observation was a result of statin-induced neutrophil sensitization and a consequential response to a lower threshold of ROS, leading to the production of NETs [85]. However, statins’ beneficial effect on the innate immune capacity of phagocytic cells against human pathogens was also described, hence, leading to the possible assumption that NETs increase might have a protective effect [85]. Consistent with the previous results are the findings of another murine and human neutrophils model in which simvastatin and mevastatin triggered higher NETs formation compared to negative controls independently from oxygen supply by depleting intracellular cholesterol from isolated neutrophils [86]. In a cohort study, significant associations were observed between MPO–DNA and HDL-C, age, history of CVD, and use of lipid-lowering drugs [87]. However, there was no mention of the effect of specific lipid-lowering therapies on NETs [87]. Further experimental studies show that administration of cholesterol particles induces the formation of NETs, while pretreatment of cells with atorvastatin significantly reduces their production [88]. In contrast, in a small prospective study of diabetic patients (*n* = 25), statins could not influence NETs formation. Though they caused a non-significant reduction in all three NETosis biomarkers that were quantified [89]. Interestingly, a clinical protocol recruiting participants of the multicenter cohort Plaque At RISK (PARISK) study, examined atherosclerotic plaque vulnerability index association with circulating NETosis biomarkers in statin-naïve (*n* = 72) and statin-treated (*n* = 109) patients [90]. A significant association was observed in plasma MPO–DNA complexes and the vulnerability index in the statin-naïve subgroup (OR = 2.08, 95% CI 1.04–4.17) with more vulnerability characteristics present in those with higher NETs levels, whereas in statin-treated population no significant association was found (OR = 1.10, 95% CI 0.68–1.79) [90]. This could support the hypothesis that statins enhance plaque stability either via a mechanism independent of NETs production or by affecting NETs release or function per se. In the post-pandemic era, it is reasonable to investigate every potential benefit a treatment can provide in the field of infectious diseases and hospitalization. A pilot study of 62 hospitalized community-acquired pneumonia with sepsis patients receiving high dose of add-on simvastatin or placebo in standard treatment for 7 days demonstrated a significant result on treatment day 4 in favor of statin administration to attenuate neutrophil susceptibility to NETosis-priming stimuli (*p* = 0.034) [91]. Moreover, in a murine model of severe asthma, simvastatin administration appeared to inhibit NETs formation in bronchoalveolar lavage and lung tissue via reduction in PAD4 expression [92].

Regarding other hypolipidemic regimens, in a murine model of null PCSK9 mice, leucocyte accumulation and NETs formation at the site of thrombosis were notably less than in wild-type controls [93]. That was attributed to the reduced expression of the leukocyte chemoattractant molecule CXCL1 in the PCSK9-deficient mice [93]. Inhibition of PCSK9 is also thought to enhance autophagy and thereby reduce oxidative stress and inflammation [94]. This mechanism could possibly affect the production of NETs. On the other hand, in FH patients, a 6-month treatment with PCSK9i reduced neutrophil count (NC) [95]. The pre-specified safety analysis from ORION-1 evaluating immune cells did not show any neutrophil alteration under PCSK9 siRNA treatment with inclisiran for 6 months [96]. To the best of our knowledge, there are no studies on the role of currently used PCSK9 inhibitors, either monoclonal antibodies evolocumab and alirocumab or synthetic small interfering RNA (siRNA) inclisiran [97] on NETs. There are also no data for the role of other cholesterol-lowering agents, namely ezetimibe and bembedoic acid, nor for triglyceride-lowering eicosapentaenoic acid (EPA) and fibrates [97] on NETs and NETosis. Similar findings for the novel triglyceride-lowering volanesorsen, an antisense oligonucleotide (ASO) that targets apoC3 mRNA; evinacumab, an angiopoietin-like 3 (ANGPTL3) inhibitor that favors lipoprotein lipase (LPL) and endothelial lipase activity; and vupanorsen, a modified ASO that targets ANGPTL3 mRNA [97]. Finally, the effect of lipoprotein(a) [Lp(a)]-lowering drugs such as pelacarsen, an ASO that targets apo(a), or olpasiran, an siRNA that targets LPA gene [97], on NETs and NETosis remain obscure.

**Figure 1:**
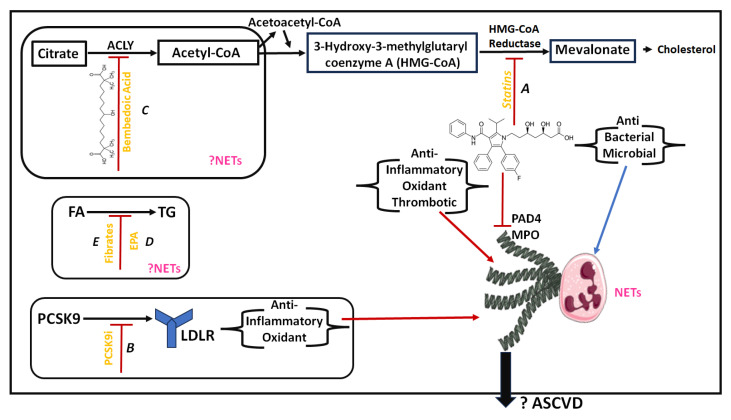
Effect of lipid-lowering therapies on NETs. A. Cholesterol synthesis and HMG CoA reductase inhibitors (statins). The pleiotropic effects of statins are described: statins have antibacterial and antimicrobial effects and could promote the production of NETs. Statins could also reduce clinical progression of sepsis. In sepsis, NETs function could be a useful biomarker. The anti-inflammatory, antioxidant, and antithrombotic effect of statins could reduce the formation of NETs via the inhibition of PAD4 and MPO. B. PSCK9 inhibitors possibly could inhibit the formation of NETs. C, D, and E. Bempedoic acid, EPA, and fibrates, respectively, with no known effect on NETs and NETosis. Black arrows indicate reaction, red arrows indicate decrease, blue arrows indicate increase, blunt arrows indicate inhibition.

Possible effects of lipid-lowering medications on NETs are summarized in Figure 1.

A summary of clinical and experimental studies that address the effects of current hypolipidemic regimens on NETs and NETosis is provided in Table 1.

## 5. Conclusions

NETs comprise an undoubtedly fascinating and promising field of research in the physiology, pathophysiology, therapeutic options, diagnosis, and monitoring of several diseases. However, the extent of NETs involvement in various conditions, like atherosclerosis, and the potential therapeutic benefits remain inadequately charted. NETs contribution to the atherosclerotic process and CVD complications is now well-documented, hence, making them an attractive area of research and a possible treatment target. Surprisingly little is known about the effect of lipid-lowering medication on NETs in the context of their pleiotropic actions. The current literature consists mostly of experimental data and few clinical studies with small sample sizes, rendering any attempt for a solid assumption precarious. However, there is a possible trend towards attenuation of NETosis and inhibition of NETs production, at least from statin treatment. Hypolipidemic medications, statins first and foremost, are the cornerstone of atherosclerosis prevention with many pleotropic effects accounting for the documented benefits. In this context, it is necessary to conduct more studies to draw safer conclusions about the interplay between statins, inflammation, and NETosis. Also, novel hypolipidemic treatments should be investigated for beneficial mechanisms of action beyond cholesterol lowering. The development of targeted treatments focusing on NETs and NETosis is an upcoming field of research that will hopefully provide better understanding of many pathologic processes, such as atherosclerosis.

## Figures and Tables

**Table 1:** Studies of the effects current hypolipidemic regimens exert on NETs and NETosis.

Authors	Year	Type of Study	Results
Chow et al. [85]	2010	Experimental	Mevastatin, lovastatin, simvastatin, fluvastatin enhance NETs production of human neutrophils in vitro.
Kessinger et al. [83]	2015	Experimental	Atorvastatin compared to PBS reduced levels of neutrophils and CitH3 in murine thrombi.
Al-Ghoul et al. [84]	2014	Experimental	Simvastatin exerted protective effect against inflammation and systemic NETosis post-thermal injury.
Liu et al. [88]	2014	Experimental	Pretreatment with atorvastatin alleviated the cholesterol-induced NETs production in vitro.
Park et al. [89]	2018	Clinical	NETosis biomarkers (NE, DNA–histone complexes, cell-free DNA) levels decreased non-significantly after 3 month treatment with moderate intensity statins in 25 diabetic patients.
Wang et al. [93]	2017	Experimental	NETs formation and leucocyte accumulation significantly reduced in PCSK9 -/- mice compared to wild-type controls.
De Vries et al. [90]	2022	Clinical	Attenuation of plasma NETosis components association with atherosclerotic plaque vulnerability index probably via effects in NETs levels or functions.
Henneck et al. [86]	2022	Experimental	Simvastatin and mevastatin trigger NETs formation in isolated neutrophils by depleting intracellular cholesterol independently from oxygen supply.
Sapey et al. [91]	2019	Clinical	Add-on high dose simvastatin versus placebo on 62 patients with community-acquired pneumonia with sepsis reduced NETosis on treatment day 4.
Chen et al. [92]	2023	Experimental	Simvastatin reduced NETs formation in bronchoalveolar lavage and lung tissue in a murine model of severe asthma.

## References

[1] Fuchs T.A., Abed U., Goosmann C., Hurwitz R., Schulze I., Wahn V., Weinrauch Y., Brinkmann V., Zychlinsky A. (2007). Novel cell death program leads to neutrophil extracellular traps. J. Cell Biol..

[2] Pan Q., Chen C., Yang Y.J. (2023). Top Five Stories of the Cellular Landscape and Therapies of Atherosclerosis: Current Knowledge and Future Perspectives. Curr. Med. Sci..

[3] Klopf J., Brostjan C., Eilenberg W., Neumayer C. (2021). Neutrophil extracellular traps and their implications in cardiovascular and inflammatory disease. Int. J. Mol. Sci..

[4] Papayannopoulos V. (2018). Neutrophil extracellular traps in immunity and disease. Nat. Rev. Immunol..

[5] Thammavongsa V., Missiakas D.M., Schneewind O. (2013). *Staphylococcus aureus* degrades neutrophil extracellular traps to promote immune cell death. Science.

[6] Barnes B.J., Adrover J.M., Baxter-Stoltzfus A., Borczuk A., Cools-Lartigue J., Crawford J.M., Daßler-Plenker J., Guerci P., Huynh C., Knight J.S. (2020). Targeting potential drivers of COVID-19: Neutrophil extracellular traps. J. Exp. Med..

[7] Pedersen S.F., Ho Y.C. (2020). SARS-CoV-2: A storm is raging. J. Clin. Investig..

[8] Zuo Y., Yalavarthi S., Shi H., Gockman K., Zuo M., Madison J.A., Blair C., Weber A., Barnes B.J., Egeblad M. (2020). Neutrophil extracellular traps in COVID-19. JCI Insight.

[9] Leppkes M., Knopf J., Naschberger E., Lindemann A., Singh J., Herrmann I., Stürzl M., Staats L., Mahajan A., Schauer C. (2020). Vascular occlusion by neutrophil extracellular traps in COVID-19. EBioMedicine.

[10] Döring Y., Libby P., Soehnlein O. (2020). Neutrophil Extracellular Traps Participate in Cardiovascular Diseases: Recent Experimental and Clinical Insights. Circ. Res..

[11] Shimony A., Zahger D., Gilutz H., Goldstein H., Orlov G., Merkin M., Shalev A., Ilia R., Douvdevani A. (2010). Cell free DNA detected by a novel method in acute ST-elevation myocardial infarction patients. Acute Card. Care.

[12] Cui M., Fan M., Jing R., Wang H., Qin J., Sheng H., Wang Y., Wu X., Zhang L., Zhu J. (2013). Cell-free circulating DNA: A new biomarker for the acute coronary syndrome. Cardiology.

[13] Meher A.K., Spinosa M., Davis J.P., Pope N., Laubach V.E., Su G., Serbulea V., Leitinger N., Ailawadi G., Upchurch G.R. (2018). Novel Role of IL (Interleukin)-1β in Neutrophil Extracellular Trap Formation and Abdominal Aortic Aneurysms. Arterioscler. Thromb. Vasc. Biol..

[14] Delbosc S., Alsac J.-M., Journe C., Louedec L., Castier Y., Bonnaure-Mallet M., Ruimy R., Rossignol P., Bouchard P., Michel J.-B. (2011). Porphyromonas gingivalis Participates in Pathogenesis of Human Abdominal Aortic Aneurysm by Neutrophil Activation. Proof of Concept in Rats. PLoS ONE.

[15] Yan H., Zhou H.F., Akk A., Hu Y., Springer L.E., Ennis T.L., Pham C.T.N. (2016). Neutrophil Proteases Promote Experimental Abdominal Aortic Aneurysm via Extracellular Trap Release and Plasmacytoid Dendritic Cell Activation. Arterioscler. Thromb. Vasc. Biol..

[16] Zenaro E., Pietronigro E., Della Bianca V., Piacentino G., Marongiu L., Budui S., Turano E., Rossi B., Angiari S., Dusi S. (2015). Neutrophils promote Alzheimer’s disease-like pathology and cognitive decline via LFA-1 integrin. Nat. Med..

[17] Arnett D.K., Blumenthal R.S., Albert M.A., Buroker A.B., Goldberger Z.D., Hahn E.J., Himmelfarb C.D., Khera A., Lloyd-Jones D., McEvoy J.W. (2019). 2019 ACC/AHA Guideline on the Primary Prevention of Cardiovascular Disease: A Report of the American College of Cardiology/American Heart Association Task Force on Clinical Practice Guidelines, Health Research Alliance manuscript submission. Circulation.

[18] Libby P. (2021). The changing landscape of atherosclerosis. Nature.

[19] Hong Y.M. (2010). Atherosclerotic Cardiovascular Disease Beginning in Childhood. Korean Circ. J..

[20] Libby P., Buring J.E., Badimon L., Hansson G.K., Deanfield J., Bittencourt M.S., Tokgözoğlu L., Lewis E.F. (2019). Atherosclerosis. Nat. Rev. Dis. Prim..

[21] Mundi S., Massaro M., Scoditti E., Carluccio M.A., Van Hinsbergh V.W.M., Iruela-Arispe M.L., De Caterina R. (2018). Endothelial permeability, LDL deposition, and cardiovascular risk factors—A review. Cardiovasc. Res..

[22] Nordestgaard B.G., Varbo A. (2014). Triglycerides and cardiovascular disease. Lancet.

[23] Tokgözoǧlu L., Libby P. (2022). The dawn of a new era of targeted lipid-lowering therapies. Eur. Heart J..

[24] Koutsogianni A.D., Liamis G., Liberopoulos E., Adamidis P.S., Florentin M. (2023). Effects of Lipid-Modifying and Other Drugs on Lipoprotein(a) Levels—Potent Clinical Implications. Pharmaceuticals.

[25] Hoogeveen R.C., Ballantyne C.M. (2021). Residual Cardiovascular Risk at Low LDL: Remnants, Lipoprotein(a), and Inflammation. Clin. Chem..

[26] Soehnlein O., Libby P. (2021). Targeting inflammation in atherosclerosis—From experimental insights to the clinic. Nat. Rev. Drug Discov..

[27] Weber C., Noels H. (2011). Atherosclerosis: Current pathogenesis and therapeutic options. Nat. Med..

[28] Brinkmann V., Reichard U., Goosmann C., Fauler B., Uhlemann Y., Weiss D.S., Weinrauch Y., Zychlinsky A. (2004). Neutrophil Extracellular Traps Kill Bacteria. Science.

[29] Saffarzadeh M., Juenemann C., Queisser M.A., Lochnit G., Barreto G., Galuska S.P., Lohmeyer J., Preissner K.T. (2012). Neutrophil Extracellular Traps Directly Induce Epithelial and Endothelial Cell Death: A Predominant Role of Histones. PLoS ONE.

[30] Moschonas I.C., Tselepis A.D. (2019). The pathway of neutrophil extracellular traps towards atherosclerosis and thrombosis. Atherosclerosis.

[31] Welin A., Amirbeagi F., Christenson K., Björkman L., Björnsdottir H., Forsman H., Dahlgren C., Karlsson A., Bylund J. (2013). The Human Neutrophil Subsets Defined by the Presence or Absence of OLFM4 Both Transmigrate into Tissue In Vivo and Give Rise to Distinct NETs In Vitro. PLoS ONE.

[32] Brinkmann V., Zychlinsky A. (2012). Neutrophil extracellular traps: Is immunity the second function of chromatin?. J. Cell Biol..

[33] Kenny E.F., Herzig A., Ger R.K., Muth A., Mondal S., Thompson P.R., Brinkmann V., Von Bernuth H., Zychlinsky A. (2017). Diverse stimuli engage different neutrophil extracellular trap pathways. Elife.

[34] Pilsczek F.H., Salina D., Poon K.K.H., Fahey C., Yipp B.G., Sibley C.D., Robbins S.M., Green F.H.Y., Surette M.G., Sugai M. (2010). A Novel Mechanism of Rapid Nuclear Neutrophil Extracellular Trap Formation in Response to *Staphylococcus aureus*. J. Immunol..

[35] Yipp B.G., Petri B., Salina D., Jenne C.N., Scott B.N.V., Zbytnuik L.D., Pittman K., Asaduzzaman M., Wu K., Meijndert H.C. (2012). Infection-induced NETosis is a dynamic process involving neutrophil multitasking in vivo. Nat. Med..

[36] Saitoh T., Komano J., Saitoh Y., Misawa T., Takahama M., Kozaki T., Uehata T., Iwasaki H., Omori H., Yamaoka S. (2012). Neutrophil extracellular traps mediate a host defense response to human immunodeficiency virus-1. Cell Host Microbe.

[37] Abdallah D.S.A., Lin C., Ball C.J., King M.R., Duhamel G.E., Denkers E.Y. (2012). Toxoplasma gondii triggers release of human and mouse neutrophil extracellular traps. Infect. Immun..

[38] Urban C.F., Reichard U., Brinkmann V., Zychlinsky A. (2006). Neutrophil extracellular traps capture and kill Candida albicans and hyphal forms. Cell. Microbiol..

[39] Kaplan M.J., Radic M. (2012). Neutrophil Extracellular Traps: Double-Edged Swords of Innate Immunity. J. Immunol..

[40] Pieterse E., Jeremic I., Czegley C., Weidner D., Biermann M.H.C., Veissi S., Maueröder C., Schauer C., Bilyy R., Dumych T. (2016). Blood-borne phagocytes internalize urate microaggregates and prevent intravascular NETosis by urate crystals. Sci. Rep..

[41] Kessenbrock K., Krumbholz M., Schönermarck U., Back W., Gross W.L., Werb Z., Gröne H.J., Brinkmann V., Jenne D.E. (2009). Netting neutrophils in autoimmune small-vessel vasculitis. Nat. Med..

[42] Thålin C., Lundström S., Seignez C., Daleskog M., Lundström A., Henriksson P., Helleday T., Phillipson M., Wallén H., Demers M. (2018). Citrullinated histone H3 as a novel prognostic blood marker in patients with advanced cancer. PLoS ONE.

[43] Borissoff J.I., Joosen I.A., Versteylen M.O., Brill A., Fuchs T.A., Savchenko A.S., Gallant M., Martinod K., Cate H.T., Hofstra L. (2013). Elevated levels of circulating DNA and chromatin are independently associated with severe coronary atherosclerosis and a prothrombotic state, Arterioscler. Thromb. Vasc. Biol..

[44] Lood C., Blanco L.P., Purmalek M.M., Carmona-Rivera C., De Ravin S.S., Smith C.K., Malech H.L., Ledbetter J.A., Elkon K.B., Kaplan M.J. (2016). Neutrophil extracellular traps enriched in oxidized mitochondrial DNA are interferogenic and contribute to lupus-like disease. Nat. Med..

[45] Adrover J.M., Aroca-Crevillén A., Crainiciuc G., Ostos F., Rojas-Vega Y., Rubio-Ponce A., Cilloniz C., Bonzón-Kulichenko E., Calvo E., Rico D. (2020). Programmed ‘disarming’ of the neutrophil proteome reduces the magnitude of inflammation. Nat. Immunol..

[46] Obama T., Itabe H. (2020). Neutrophils as a Novel Target of Modified Low-Density Lipoproteins and an Accelerator of Cardiovascular Diseases. Int. J. Mol. Sci..

[47] Masuda S., Nakazawa D., Shida H., Miyoshi A., Kusunoki Y., Tomaru U., Ishizu A. (2016). NETosis markers: Quest for specific, objective, and quantitative markers. Clin. Chim. Acta.

[48] Pantazi D., Tellis C., Tselepis A.D. (2022). Oxidized phospholipids and lipoprotein-associated phospholipase A2 (Lp-PLA2) in atherosclerotic cardiovascular disease: An update. Biofactors.

[49] Hansen S.E.J., Madsen C.M., Varbo A., Nordestgaard B.G. (2019). Low-Grade Inflammation in the Association between Mild-to-Moderate Hypertriglyceridemia and Risk of Acute Pancreatitis: A Study of More Than 115000 Individuals from the General Population. Clin. Chem..

[50] Varbo A., Benn M., Tybjærg-Hansen A., Nordestgaard B.G. (2013). Elevated remnant cholesterol causes both low-grade inflammation and ischemic heart disease, whereas elevated low-density lipoprotein cholesterol causes ischemic heart disease without inflammation. Circulation.

[51] Ridker P.M. (2016). A Test in Context: High-Sensitivity C-Reactive Protein. J. Am. Coll. Cardiol..

[52] Xiao L., Harrison D.G. (2020). Inflammation in Hypertension. Can. J. Cardiol..

[53] Ross R., Neeland I.J., Yamashita S., Shai I., Seidell J., Magni P., Santos R.D., Arsenault B., Cuevas A., Hu F.B. (2020). Waist circumference as a vital sign in clinical practice: A Consensus Statement from the IAS and ICCR Working Group on Visceral Obesity. Nat. Rev. Endocrinol..

[54] Giovenzana A., Carnovale D., Phillips B., Petrelli A., Giannoukakis N. (2021). Neutrophils and their role in the aetiopathogenesis of type 1 and type 2 diabetes. Diabetes. Metab. Res. Rev..

[55] Libby P., Hansson G.K. (2019). From Focal Lipid Storage to Systemic Inflammation: JACC Review Topic of the Week. J. Am. Coll. Cardiol..

[56] Paulson K.E., Zhu S.N., Chen M., Nurmohamed S., Jongstra-Bilen J., Cybulsky M.I. (2010). Resident intimal dendritic cells accumulate lipid and contribute to the initiation of atherosclerosis. Circ. Res..

[57] Lim H.Y., Lim S.Y., Tan C.K., Thiam C.H., Goh C.C., Carbajo D., Chew S.H.S., See P., Chakarov S., Wang X.N. (2018). Hyaluronan Receptor LYVE-1-Expressing Macrophages Maintain Arterial Tone through Hyaluronan-Mediated Regulation of Smooth Muscle Cell Collagen. Immunity.

[58] Owsiany K.M., Alencar G.F., Owens G.K. (2019). Revealing the origins of foam cells in atherosclerotic lesions. Arterioscler. Thromb. Vasc. Biol..

[59] Sheedy F.J., Grebe A., Rayner K.J., Kalantari P., Ramkhelawon B., Carpenter S.B., Becker C.E., Ediriweera H.N., Mullick A.E., Golenbock D.T. (2013). CD36 coordinates NLRP3 inflammasome activation by facilitating intracellular nucleation of soluble ligands into particulate ligands in sterile inflammation. Nat. Immunol..

[60] Westerterp M., Fotakis P., Ouimet M., Bochem A.E., Zhang H., Molusky M.M., Wang W., Abramowicz S., La Bastide-Van Gemert S., Wang N. (2018). Cholesterol efflux pathways suppress inflammasome activation, NETosis, and atherogenesis. Circulation.

[61] Kahlenberg J.M., Carmona-Rivera C., Smith C.K., Kaplan M.J. (2013). Neutrophil extracellular trap-associated protein activation of the NLRP3 inflammasome is enhanced in lupus macrophages. J. Immunol..

[62] Paulin N., Viola J.R., Maas S.L., De Jong R., Fernandes-Alnemri T., Weber C., Drechsler M., Döring Y., Soehnlein O. (2018). Double-Strand DNA Sensing Aim2 Inflammasome Regulates Atherosclerotic Plaque Vulnerability. Circulation.

[63] Libby P. (2013). Collagenases and cracks in the plaque. J. Clin. Investig..

[64] Davies M.J. (1996). Stability and instability: Two faces of coronary atherosclerosis. The Paul Dudley White Lecture 1995. Circulation.

[65] Douglas P.S., Hoffmann U., Patel M.R., Mark D.B., Al-Khalidi H.R., Cavanaugh B., Cole J., Dolor R.J., Fordyce C.B., Huang M. (2015). Outcomes of anatomical versus functional testing for coronary artery disease. N. Engl. J. Med..

[66] (2018). Coronary CT Angiography and 5-Year Risk of Myocardial Infarction. N. Engl. J. Med..

[67] Stone G.W., Maehara A., Lansky A.J., de Bruyne B., Cristea E., Mintz G.S., Mehran R., McPherson J., Farhat N., Marso S.P. (2011). A prospective natural-history study of coronary atherosclerosis. N. Engl. J. Med..

[68] Franck G., Even G., Gautier A., Salinas M., Loste A., Procopio E., Gaston A.T., Morvan M., Dupont S., Deschildre C. (2019). Haemodynamic stress-induced breaches of the arterial intima trigger inflammation and drive atherogenesis. Eur. Heart J..

[69] Libby P. (2019). Once more unto the breach: Endothelial permeability and atherogenesis. Eur. Heart J..

[70] Molinaro R., Yu M., Sausen G., Bichsel C.A., Corbo C., Folco E.J., Lee G.Y., Liu Y., Tesmenitsky Y., Shvartz E. (2021). Targeted delivery of protein arginine deiminase-4 inhibitors to limit arterial intimal NETosis and preserve endothelial integrity. Cardiovasc. Res..

[71] Megens R.T.A., Vijayan S., Lievens D., Döring Y., van Zandvoort M.A.M.J., Grommes J., Weber C., Soehnlein O. (2012). Presence of luminal neutrophil extracellular traps in atherosclerosis. Thromb. Haemost..

[72] Pertiwi K.R., Van Der Wal A.C., Pabittei D.R., Mackaaij C., Van Leeuwen M.B., Li X., De Boer O.J. (2018). Neutrophil Extracellular Traps Participate in All Different Types of Thrombotic and Haemorrhagic Complications of Coronary Atherosclerosis. Thromb. Haemost..

[73] Silvestre-Roig C., Braster Q., Wichapong K., Lee E.Y., Teulon J.M., Berrebeh N., Winter J., Adrover J.M., Santos G.S., Froese A. (2019). Externalized histone H4 orchestrates chronic inflammation by inducing lytic cell death. Nature.

[74] Warnatsch A., Ioannou M., Wang Q., Papayannopoulos V. (2015). Inflammation. Neutrophil extracellular traps license macrophages for cytokine production in atherosclerosis. Science.

[75] Franck G., Mawson T.L., Folco E.J., Molinaro R., Ruvkun V., Engelbertsen D., Liu X., Tesmenitsky Y., Shvartz E., Sukhova G.K. (2018). Roles of PAD4 and NETosis in Experimental Atherosclerosis and Arterial Injury: Implications for Superficial Erosion. Circ. Res..

[76] Knight J.S., Luo W., O’Dell A.A., Yalavarthi S., Zhao W., Subramanian V., Guo C., Grenn R.C., Thompson P.R., Eitzman D.T. (2014). Peptidylarginine Deiminase Inhibition Reduces Vascular Damage and Modulates Innate Immune Responses in Murine Models of Atherosclerosis. Circ. Res..

[77] Liu Y., Carmona-Rivera C., Moore E., Seto N.L., Knight J.S., Pryor M., Yang Z.H., Hemmers S., Remaley A.T., Mowen K.A. (2018). Myeloid-Specific Deletion of Peptidylarginine Deiminase 4 Mitigates Atherosclerosis. Front. Immunol..

[78] Rohrbach A.S., Hemmers S., Arandjelovic S., Corr M., Mowen K.A. (2012). PAD4 is not essential for disease in the K/BxN murine autoantibody-mediated model of arthritis. Arthritis Res. Ther..

[79] Badimon L., Vilahur G. (2015). Neutrophil extracellular traps: A new source of tissue factor in atherothrombosis. Eur. Heart J..

[80] Chirivi R.G.S., van Rosmalen J.W.G., van der Linden M., Euler M., Schmets G., Bogatkevich G., Kambas K., Hahn J., Braster Q., Soehnlein O. (2020). Therapeutic ACPA inhibits NET formation: A potential therapy for neutrophil-mediated inflammatory diseases. Cell. Mol. Immunol..

[81] Vogel B., Shinagawa H., Hofmann U., Ertl G., Frantz S. (2015). Acute DNase1 treatment improves left ventricular remodeling after myocardial infarction by disruption of free chromatin. Basic Res. Cardiol..

[82] Ge L., Zhou X., Ji W.J., Lu R.Y., Zhang Y., Zhang Y.D., Ma Y.Q., Zhao J.H., Li Y.M. (2015). Neutrophil extracellular traps in ischemia-reperfusion injury-induced myocardial no-reflow: Therapeutic potential of DNase-based reperfusion strategy. Am. J. Physiol. Heart Circ. Physiol..

[83] Kessinger C.W., Kim J.W., Henke P.K., Thompson B., McCarthy J.R., Hara T., Sillesen M., Margey R.J.P., Libby P., Weissleder R. (2015). Statins improve the resolution of established murine venous thrombosis: Reductions in thrombus burden and vein wall scarring. PLoS ONE.

[84] Al-Ghoul W.M., Kim M.S., Fazal N., Azim A.C., Ali A. (2014). Evidence for simvastatin anti-inflammatory actions based on quantitative analyses of NETosis and other inflammation/oxidation markers. Results Immunol..

[85] Chow O.A., Von Köckritz-Blickwede M., Bright A.T., Hensler M.E., Zinkernagel A.S., Cogen A.L., Gallo R.L., Monestier M., Wang Y., Glass C.K. (2010). Statins enhance formation of phagocyte extracellular traps. Cell Host Microbe.

[86] Henneck T., Mergani A., Clever S., Seidler A.E., Brogden G., Runft S., Baumgärtner W., Branitzki-Heinemann K., von Köckritz-Blickwede M.V. (2022). Formation of Neutrophil Extracellular Traps by Reduction of Cellular Cholesterol Is Independent of Oxygen and HIF-1α. Int. J. Mol. Sci..

[87] Donkel S.J., Wolters F.J., Ikram M.A., de Maat M.P.M. (2021). Circulating Myeloperoxidase (MPO)-DNA complexes as marker for Neutrophil Extracellular Traps (NETs) levels and the association with cardiovascular risk factors in the general population. PLoS ONE.

[88] Cholesterol Loading Induces Neutrophil Extracellular Traps, and Atorvastatin Attenuates This Effect—ACR Meeting Abstracts, (n.d.). https://acrabstracts.org/abstract/cholesterol-loading-induces-neutrophil-extracellular-traps-and-atorvastatin-attenuates-this-effect/.

[89] Park H.S., Gu J.Y., Yoo H.J., Han S.E., Park C.H., Kim Y.I., Nam-Goong I.S., Kim E.S., Kim H.K. (2018). Thrombin Generation Assay Detects Moderate-Intensity Statin-Induced Reduction of Hypercoagulability in Diabetes. Clin. Appl. Thromb..

[90] de Vries J.J., Autar A.S.A., van Dam-Nolen D.H.K., Donkel S.J., Kassem M., van der Kolk A.G., van Velzen T.J., Kooi M.E., Hendrikse J., Nederkoorn P.J. (2022). Association between plaque vulnerability and neutrophil extracellular traps (NETs) levels: The Plaque at RISK study. PLoS ONE.

[91] Sapey E., Patel J.M., Greenwood H., Walton G.M., Grudzinska F., Parekh D., Mahida R.Y., Dancer R.C.A., Lugg S.T., Howells P.A. (2019). Simvastatin improves neutrophil function and clinical outcomes in pneumonia a pilot randomized controlled clinical trial. Am. J. Respir. Crit. Care Med..

[92] Chen Y.R., Xiang X.D., Sun F., Xiao B.W., Yan M.Y., Peng B., Liu D. (2023). Simvastatin Reduces NETosis to Attenuate Severe Asthma by Inhibiting PAD4 Expression. Oxid. Med. Cell. Longev..

[93] Wang H., Wang Q., Wang J., Guo C., Kleiman K., Meng H., Knight J.S., Eitzman D.T. (2017). Proprotein convertase subtilisin/kexin type 9 (PCSK9) Deficiency is Protective against Venous Thrombosis in Mice. Sci. Rep..

[94] Yang J., Ma X., Niu D., Sun Y., Chai X., Deng Y., Wang J., Dong J. (2023). PCSK9 inhibitors suppress oxidative stress and inflammation in atherosclerotic development by promoting macrophage autophagy. Am. J. Transl. Res..

[95] Scicali R., Di Pino A., Ferrara V., Rabuazzo A.M., Purrello F., Piro S. (2021). Effect of PCSK9 inhibitors on pulse wave velocity and monocyte-to-HDL-cholesterol ratio in familial hypercholesterolemia subjects: Results from a single-lipid-unit real-life setting. Acta Diabetol..

[96] Landmesser U., Haghikia A., Leiter L.A., Wright R.S., Kallend D., Wijngaard P., Stoekenbroek R., Kastelein J.J., Ray K.K. (2021). Effect of inclisiran, the small-interfering RNA against proprotein convertase subtilisin/kexin type 9, on platelets, immune cells, and immunological biomarkers: A pre-specified analysis from ORION-1. Cardiovasc. Res..

[97] Kim K., Ginsberg H.N., Choi S.H. (2022). New, Novel Lipid-Lowering Agents for Reducing Cardiovascular Risk: Beyond Statins. Diabetes Metab. J..

